# Knowledge, Attitudes, and Practices towards Influenza Vaccine among Guangzhou Residents: A Cross-Sectional Study

**DOI:** 10.3390/vaccines12101169

**Published:** 2024-10-14

**Authors:** Jiawen Xu, Jianyun Lu, Qing He, Yu Ma, Keyi Wu, Haowen Chen, Xiaowei Ma, Xianbo Wu

**Affiliations:** 1 School of Public Health, Southern Medical University, Guangzhou 510515, China; 2 School of Public Health, Sun Yat-Sen University, Guangzhou 510080, China; 3 Guangzhou Baiyun Center for Disease Control and Prevention, Guangzhou 510440, China; 4 Guangzhou Center for Disease Control and Prevention, Guangzhou 510440, China; 5 Shenzhen Nanshan District Center for Disease Control and Prevention, Shenzhen 518054, China; 6 Guangdong Provincial Key Laboratory of Tropical Disease Research, Department of Epidemiology, School of Public Health, Southern Medical University, Guangzhou 510515, China

**Keywords:** seasonal influenza vaccination, influenza vaccine willingness, vaccine hesitancy, influencing factors

## Abstract

Background: Influenza vaccination is an important prevention strategy for flu illness. However, the vaccination rate is still low in Guangzhou, China. This study aimed to understand the status of knowledge, the attitude towards the vaccines’ reliability and safety, and other aspects associated with the willingness and practice of influenza vaccines in the pediatric and adult populations of Guangzhou city. Methods: This study was performed in eleven districts in Guangzhou between November 2020 and December 2020, including the Yuexiu, Liwan, Haizhu, Tianhe, Baiyun, Panyu, Huadu, Nansha, Huangpu, Zengcheng, and Conghua districts. The parents of children and teenagers under the age of eighteen in Guangzhou were surveyed using self-administered questionnaires in four domains: demographic information, the knowledge status and perception of influenza vaccination, the willingness and attitude towards influenza vaccination, and previous vaccine uptake. A multivariable logistic regression was employed to assess the possible determinants of willingness and practice to receive influenza vaccination, calculating the odds ratios (ORs) and 95% confidence interval (CI). A two-sided *p*-value < 0.05 was deemed statistically significant. Results: A total of 13,213 valid questionnaires were collected (validity rate 98.8%). Out of these participants, 42.62% (5631 participants) expressed a willingness to receive the influenza vaccine, while 55.40% (7320 participants) reported that their children and teenagers had been vaccinated against the flu. Furthermore, 40.44% of the respondents (5343 participants) or other family members had received the influenza vaccine. Logistic regression indicated that factors such as being female (OR = 1.395, 95% CI: 1.278–1.522), being involved in the work of COVID-19 prevention and control (1.551, 1.396–1.724), affirming the preventive effects of vaccination (2.474, 2.106–2.906), knowing about annual influenza vaccination (2.756, 2.540–2.992), and understanding prioritized influenza vaccination populations (1.464, 1.343–1.596) were all positively associated with vaccination willingness. Conversely, middle-aged persons (aged 40–49 years old) (0.726, 0.617–0.853), higher educational levels (undergraduate versus middle school) (0.858, 0.768–0.959), heightened concerns about vaccine safety (considering side effects are obvious versus considering it is safe and basically no side effects) (0.284, 0.188–0.429) and lower knowledge scores (0.813, 0.701–0.942) were adversely linked with vaccination willingness. Conclusion: These findings provide essential insights for altering the perception of influence and influenza vaccination, as well as enhancing health communication strategies to improve influenza vaccine uptake among Guangzhou residents.

## 1. Introduction

Influenza is an acute respiratory infectious disease caused by the influenza virus, which is highly variable and can be divided into types A, B, C, and D [1]. It poses a serious threat to public health as it is highly contagious and spreads rapidly. Owing to the climatic characteristics in the Northern Hemisphere, influenza activity tends to escalate in October each year, peaking from December through February in the following year and presenting noticeable seasonal trends [2]. Globally, approximately one billion annual incidences of seasonal infections occur, encompassing three to five million serious cases and up to 290,000 to 650,000 respiratory-related fatalities [3,4]. In China, the annual number of cases is approximately 84 million to 144 million, resulting in an all-cause excess mortality rate due to influenza of between 6.9/100,000 and 17.2/100,100 [5].

Nowadays, vaccination has been recognized as the principal public health strategy in controlling infectious diseases and preventing disease outbreaks. Seasonal influenza vaccination (SIV) has been proven effective in mitigating the health and societal impacts of influenza by minimizing the risk of severe disease and decreasing complications and fatalities among high-risk groups, including infants and young children, pregnant women, the elderly, and individuals with chronic diseases. Both the Chinese Guidelines on Influenza Vaccine Prevention (2022–2023) and the United States Center for Disease Control and Prevention (CDC) recommend the annual influenza vaccination to all non-contraindicated residents aged six months and older [6,7]. Some major cities, such as Beijing and Shenzhen, have enacted policies offering free influenza vaccines to specific high-risk groups, including senior citizens, healthcare workers, and children [8]. However, the influenza vaccination rate is still low in many regions of China due to the limitations of residents’ knowledge, lack of public vaccine awareness, local health policies, and prevention education [9]. The disparities in influenza vaccine coverage across countries correlate with the level of knowledge and attitude toward seasonal influenza vaccines. It may also suggest that attention should be paid not only to improving its affordability but also to enhancing people’s vaccine knowledge, willingness, and practice. According to the *2021 Influenza Season Vaccination Coverage Estimation Report*, the vaccination rate of Guangdong Province is only 3.42%, significantly lower than the 80% target for adults aged 18–64 years established by the “Healthy People 2030” immunization and infectious disease goals [9,10].

In 2020, the coronavirus disease 2019 (COVID-19) outbreak contributed to a rising number of co-circulation situations with diseases such as influenza. On the one hand, this increased the risk of patients suffering from multiple infections, requiring hospitalizations and overwhelming the healthcare system [11]. On the other hand, the seriousness of COVID-19 drives interest in the influenza vaccine, prompting health-seeking behavior and increasing the rate of respiratory-related vaccination [12].

A prior study on influenza vaccination also reported a close association between vaccination practices and several elements, including vaccination decisions, public awareness, economic barriers, vaccine or disease-related knowledge, perceived vulnerability, and seriousness of diseases [13]. Nevertheless, previous cross-sectional studies have had some limitations, including recruitment of a particular population (healthcare workers and the elderly), notable regional and population differences (various cultural backgrounds, public health support, medical resources, and acceptance of vaccines), and small sample sizes. As a significant area and one of the biggest cities in Southern China, Guangzhou City reports a high incidence of influenza cases. However, the potential factors that influence Guangzhou’s children and parents’ willingness and practice toward influenza vaccination are still far from clear. Therefore, we undertook a cross-sectional study to comprehensively analyze parents’ knowledge and attitudes toward the reliability and safety of the influenza vaccine and other aspects associated with the willingness and practice of influenza vaccination. This study provides a basis for prevention and targeted interventions.

## 2. Methods

### 2.1. Study Participants

We performed a cross-sectional study from November to December of 2020 utilizing a self-administered electronic questionnaire that was conducted in Guangzhou City in Southern China, which is home to over fifteen million residents. A convenience sampling survey methodology was used to recruit permanent families from eleven districts of Guangzhou, including the Yuexiu, Liwan, Haizhu, Tianhe, Baiyun, Panyu, Huadu, Nansha, Huangpu, Zengcheng, and Conghua districts. The inclusion participants for this study were parents of children and teenagers under the age of eighteen who had lived in the selected districts for more than three months before participating in the survey. Our primary focus was on children under the age of fourteen. This population attends kindergarten, elementary, and middle schools and typically lacks developed or solidified good hygiene habits. Then, we sent the electronic questionnaires to the selected families, and the survey was conducted on Wen Juan Xing (Changsha Ranxing Information Technology Co., Ltd., Changsha, Hunan, China), the largest online survey platform in China. The parents were responsible for providing accurate information on the influenza vaccination of their children, teenagers, and their family members (not including the children and teenagers) in the past, and it was unnecessary to fill in the questionnaire repeatedly if there were multiple children and teenagers in the same family. We only collected information from one of the parents of the same family. The answers to the questionnaires were under quality control; only the respondents without logical or input errors were included successfully. Before conducting the survey, we explained its purpose to the participants and obtained informed consent from them. Participants were asked to provide information on behalf of their children, teenagers, and other family members (not including children and teenagers). The protocol for this research was approved by the ethics committee of Guangzhou CDC (GZCDC 2019021).

### 2.2. Questionnaire

The questionnaire comprised four parts, with a total of 40 questions covering baseline demographic information, the knowledge status and perception of influenza vaccination, willingness and attitude towards influenza vaccination, and the vaccine status of individuals, their children and teenagers, and their family members (not including the children and teenagers) in the past. The first part contained the socio-demographic characteristics of the parents, their children, and teenagers, such as one of the parents’ ages (aged 18 to 29 years, aged 30 to 39 years, aged 40 to 49 years, and aged ≥50 years), sex (female/male), educational level (middle school and below, high school, specialized training school, undergraduate, and postgraduate and above), whether involved in the work of prevention and control of COVID-19 (yes/no), monthly household income (<4000 yuan/m, 4000–9999) yuan/m, 10,000–19,999 yuan/m, and 20,000 and more yuan/m), residential districts (suburban/urban), number of family members (1–3 members, 4–5 members, and 6 and above members), and number of children under fourteen years old in the family (0 child, one child, and two or more children). When the participants reported that they lived in either Yuexiu, Liwan, Haizhu, Tianhe, Baiyun, Panyu, or Huangpu, we classified the living region as urban. The second part focused on the participants’ knowledge and perception of influenza and the seasonal influenza vaccine. The participants were asked, “Do you think the influenza vaccine is safe?” and the answers were categorized into “it’s safe, basically no side effects”, “basically safe, with a few side effects”, and “it’s not safe. Side effects are obvious”. We calculated a knowledge score derived from the responses to the four influenza vaccine-related questions. Each question was assigned zero points for a defined wrong answer and one point for a correct response, resulting in a maximum possible score of four points. Participants’ perceptions about the effectiveness of the vaccine were evaluated using a binary question (Yes or No): “Do you consider the influenza vaccine can prevent the flu?”. Individuals who responded “Yes” scored one point. The following assessment of participants’ knowledge about the vaccine was used: “Does the flu shot need to be given every year?” and the defined correct answer was “Yes”. The participants were also asked, “Which groups needs to be prioritized for influenza vaccination?” and the simultaneously selected “children aged six months to five years”, “seniors aged sixty years and above”, “patients with chronic illnesses”, “healthcare workers”, and “family members and caregivers of infants under six months” scored one point. The last question about knowledge of the vaccine was, “What time of year is it appropriate to get influenza vaccination in Guangzhou?” and the answer “September to December” was correct (Table S1). The third part comprised seven questions about participants’ willingness and attitude towards influenza vaccination. The participants were asked, “Are you or your family members willing to take influenza vaccine?” with two answers provided, “yes” or “no”. When the participants answered “yes”, we asked for the reasons with the reasons categorized into “the COVID-19 epidemic is serious and the fear of infection affects the normal life of the individuals”, “group vaccination organized by the workplace”, “knowing that friends and relatives in the neighborhood have vaccination plans”, and “getting the flu shot for fear of contracting the COVID-19”. In addition, the parents who had never taken their children and/or teenagers for an influenza vaccine in the past were asked, “Are you willing to start to take your own kids (including teenagers) for a flu shot this year?” and were then asked the reasons for considering or not considering. Furthermore, for the parents who had taken their children and teenagers for an influenza vaccine this year, we asked, “Are you willing to continue to take your kids for influenza vaccine in the next year?” and then asked for their reasons for continuing or not continuing. For the parents who have taken their children and teenagers for an influenza vaccine in the past, we asked for the reasons. The fourth part collected information on the individuals, their children and teenagers, and other family members (not including the children and teenagers) regarding their previous influenza vaccine status. All questions were closed-ended and treated as categorical variables (Table S2).

The Cronbach’s alpha coefficient of the questionnaire was 0.740, which indicates that the questionnaire has high internal consistency. In the process of the questionnaire’s design, a large number of existing pieces of literature and mature questionnaires were referred to so as to ensure that the content of our questionnaire was reasonable and scientific. At the same time, five experts in related fields were invited to review the questionnaire, resulting in a content validity index of 1 at the questionnaire level, which can be considered as a scale that has good content validity [14].

### 2.3. Statistical Analysis

Descriptive statistics were conducted to present the general characteristics of the participants, their knowledge and perception of influenza and vaccination, their willingness to accept the vaccination, and their practice of vaccination. The information on monthly household incomes was presented in Renminbi (RMB) (one US dollar (USD) = seven RMB). The categorical variables were described as numbers and percentages (%). Individuals’ willingness and previous history of children, teenagers, individuals, and other family members’ influenza vaccinations were the dependent variables, respectively. Bivariate analysis included one independent variable and one dependent variable for each group comparison. Multivariate analysis included a single dependent variable along with multiple independent variables calculating the odds ratios (ORs) and 95% confidence interval (CI). We applied three multivariate logistic regression models (Model 1–3) to further explore the associations of potential influencing factors with the willingness and the previous practice of influenza vaccination, respectively. Only statistically significant variables in the bivariate analysis were involved in the corresponding regression model [15]. The independent variables were selected for inclusion in the regression equation using the enter method, employing an entry probability value of 0.05 and an exclusion probability value of 0.10.

The statistical analyses were conducted using SPSS 24.0 software (IBM, New York, NY, USA). All tests were two-sided, and *p*-values < 0.05 were considered statistically significant.

## 3. Results

### 3.1. Participants’ Characteristics

A cumulative total of 13,379 electronic questionnaires had been submitted, with 13,213 of these being deemed valid for analysis after a thorough review and validation process to ensure the integrity and reliability of the collected data (validity rate 98.8%). The descriptive characteristics and their willingness to accept an influenza vaccine are shown in Table 1. The majority of participants were living in urban areas (74.72%), were female (74.18%), middle-aged between 40 and 50 (48.38%), had an undergraduate educational level (36.09%), had a household income level of four to less than ten thousand yuan (34.49%), had four to five family members (56.61%), and had one child (43.25%). Only 14.31% of the participants were used to engage in work related to the prevention and control of COVID-19 (Table 1).

### 3.2. Knowledge and Perception toward Influenza and Vaccinations

Most participants (90.56%) agreed that influenza vaccination can prevent the disease, and a large proportion of citizens (61.13%) knew that the influenza vaccine needs to be given every year. Further analysis revealed that only 33.38% of the participants answered correctly about the prior population groups needing to receive the influenza vaccine, and 30.96% knew the appropriate time to receive vaccination. A total of 28.56% of them believed the vaccine was totally safe, and 69.57% agreed that the symptoms following influenza vaccination were not severe. Moreover, only 1093 participants (8.27%) answered all the influenza vaccine-related questions correctly (Table 2 and Table S3).

### 3.3. Willingness and Attitudes towards Influenza Vaccination

A total of 5631 participants (42.62%) were willing to accept the influenza vaccination. When it came to the reasons for being willing to receive an influenza vaccine during the COVID-19 period, a striking 88.99% of the participants expressed concern about the COVID-19 outbreak as well as the severity of the impact on their personal lives and, thus, were willing to receive the vaccination. A total of 34.31% of the participants were worried about COVID-19 infection, and 27.81% of them knew that relatives and friends had vaccination plans. Only 18.06% of the participants were willing to receive vaccination because they were organized by the workplace (Table S2).

### 3.4. Practice towards Influenza Vaccination

A total of 7320 (55.40%) participants’ children and teenagers under eighteen years old had received a previous influenza vaccination, with 36.26% receiving it only once. A total of 45.41% were vaccinated spontaneously, 32.98% were vaccinated on the advice of a doctor, and 18.85% were vaccinated as a school requirement. Among the parents who took their children and teenagers for vaccination last year, 62.66% intend to continue vaccinating their children and teenagers against influenza this year. Of those, 58.13% thought vaccination could prevent the infection of other family members, and 47.25% thought it was very effective and that children and teenagers rarely contracted flu after vaccination. Parents who had previously taken their children and teenagers for flu vaccination often discontinued this practice primarily due to the thoughts that flu symptoms are not severe. A total of 48.55% of parents started to take their children and teenagers for influenza vaccination this year. The main reason why they expressed their reluctance toward vaccination was safety (Table 3 and Table S2).

A total of 5343 (40.44%) participants or their family members (not children and teenagers) had received vaccination in the past. Those who had received vaccination were more likely female, middle-aged between 40–50, had an undergraduate education, were not engaging in work related to the prevention and control of COVID-19, had a household income level of four to less than ten thousand yuan, had four to five family members, had one child under fourteen years old, and knew about influenza and the vaccine (Table 3).

### 3.5. Factors Associated with Willingness to Receive Influenza Vaccination

In the multivariate logistic regression of parents’ individual willingness, compared to males, females were more willing to be vaccinated (OR 1.395, 95% CI: 1.278–1.522). Compared to those aged 18–29 years, participants aged 30 years and older were related to negative attitudes toward influenza vaccination. Participants with undergraduate educational levels also exhibited a lower willingness to get vaccinated (OR 0.858, 95% CI: 0.768–0.959). Participants who engaged in work related to the prevention and control of COVID-19 had a greater willingness to receive vaccination (OR 1.551, 95% CI: 1.396–1.724). We further investigated the influencing factors associated with a willingness to receive an influenza vaccine, which relates to knowledge and perceptions of influenza and vaccinations. Participants who agreed that influenza vaccination is sufficient to prevent flu (OR 2.474, 95% CI: 2.106–2.906) and influenza vaccine needs to be given every year (OR 2.756, 95% CI: 2.540–2.992) were related to the positive attitude toward influenza vaccination. In parallel, the knowledge about the prior population groups needing to receive the influenza vaccine was also associated with greater willingness toward vaccination (OR 1.464, 95% CI: 1.343–1.596). The concern about the safety of vaccines was negatively associated with the willingness toward vaccination (OR 0.284, 95% CI: 0.188–0.429). Participants who did not know about influenza and vaccination were more likely to yield lower willingness toward vaccination (OR 0.813, 95% CI: 0.701–0.942) (Table 4).

### 3.6. Factors Associated with Practice of Influenza Vaccination

Regarding children’s vaccinations, mothers comparatively wielded a stronger negative influence on childhood influenza vaccination than fathers (OR = 0.911, 95% CI: 0.837–0.991). More children under the age of fourteen in the family tended to have a more positive influence on the patterns of children’s and teenagers’ previous influenza vaccinations (one child: OR = 1.389, 95% CI: 1.245–1.550; two or more children: OR = 1.474, 95% CI: 1.295–1.678). Parents who acknowledged the preventive capability of the influenza vaccine (OR = 1.438, 95% CI: 1.265–1.635), concurred with the annual administration of the influenza vaccine (OR = 1.369, 95% CI: 1.268–1.478), and were aware of the prior vaccination demographics (OR = 1.136, 95% CI: 1.045–1.235) tended to foster positive vaccination habits in children. Conversely, parents who attained specialty (OR = 0.798, 95% CI: 0.704–0.906), undergraduate (OR = 0.649, 95% CI: 0.570–0.738), or postgraduate education (OR = 0.534, 95% CI: 0.444–0.642) were observed to influence children towards negative vaccination behaviors in contrast to parents with only a middle school education. Households earning a monthly income of either ten to less than twenty thousand (OR = 0.761, 95% CI: 0.670–0.864) or twenty and exceeding twenty thousand (OR = 0.795, 95% CI: 0.693–0.912) negatively impacted children’s vaccination behaviors in comparison to those earning under four thousand. Parents who harbored skepticism about the safety of the influenza vaccine (OR = 0.320, 95% CI: 0.238–0.430) similarly exerted a negative influence on child vaccination behaviors. Parents who did not know about influenza and vaccination were more likely to yield a negative impact on children’s vaccinations (OR 0.824, 95% CI: 0.711–0.955) (Table 4).

Regarding the previous vaccination behavior of individuals or other family members, females were found to exert a more positive influence compared to males (OR = 1.366, 95% CI: 1.257–1.485). Furthermore, those who engaged in jobs related to the prevention and control of COVID-19 (OR = 1.362, 95% CI: 1.231–1.507), individuals with children under the age of fourteen (one child: OR = 1.303, 95% CI: 1.164–1.460; two or more children: OR = 1.336, 95% CI: 1.171–1.525), and those believing that the influenza vaccine protects against influenza (OR = 1.509, 95% CI: 1.313–1.734) also positively influenced vaccination behavior. In addition, those participants agreeing that the influenza vaccine is effective (OR = 1.509, 95% CI: 1.313–1.734), with the knowledge that the influenza vaccine needs to be given annually (OR = 1.535, 95% CI: 1.408–1.648) and who correctly answered which population groups should be prioritized for vaccinations (OR = 1.224, 95% CI: 1.126–1.331) were also factors facilitating vaccination. On the other hand, the age group of 30–39 years old (OR = 0.811, 95% CI: 0.694–0.948) and 40–49 years old (OR = 0.788, 95% CI: 0.674–0.920), compared to those 18–29 years old, were found to exert a negative influence on vaccination behavior. Similarly, those with an educational level of specialty, high school (OR = 0.875, 95% CI: 0.772–0.991), college (OR = 0.847, 95% CI: 0.747–0.959), or postgraduate (OR = 0.713, 95% CI: 0.594–0.857) were more likely to have an inhibitory effect on vaccination behavior when compared to those with only a middle school education. Mass distrust of influenza vaccine safety also decreased the likelihood of vaccination (OR = 0.526, 95% CI: 0.384–0.722) (Table 4).

## 4. Discussion

Influenza vaccination has proven effective in preventing infections [16]. The World Health Organization (WHO) stresses the importance of consistent influenza management amidst resource redistribution and heightened attention towards COVID-19, anticipating that the forthcoming years will witness overlaps between the spread of COVID-19 and seasonal influenza. During this study period, effective COVID-19 vaccines had not been disseminated, global severe illnesses and deaths were increasing, influenza vaccines and antivirals were readily available, and our understanding of influenza virus transmission dynamics had developed. Consequently, this study investigated the factors influencing influenza vaccination intentions and practices, proposing useful recommendations for maintaining influenza management during the ongoing COVID-19 pandemic.

### 4.1. Attitudes and Practices toward Influenza Vaccination during the COVID-19 Epidemic

In this study, we discovered that participation in works related to the mitigation and control of the ongoing COVID-19 epidemic had a significant positive impact on an individual’s willingness and behavior toward influenza vaccination for themselves and other family members (excluding children and teenagers). A cross-sectional study from the Czech Republic revealed that during the 2020–2021 influenza season, health professionals were more inclined to take the influenza vaccine due to concerns about the concurrent risks of influenza and COVID-19 [17]. Another cross-sectional study reported that approximately 20% of Canadians aged 50 to 64 who initially planned not to have the influenza vaccine reconsidered their decision and had planned to take the vaccine during the 2020–2021 season due to the COVID-19 epidemic [18]. Furthermore, a Canadian cohort study presented pieces of evidence showing that influenza vaccination behavior corresponded strongly with the willingness to be vaccinated against COVID-19 infection [19]. During the COVID-19 epidemic, coverage of the influenza vaccine increased across ten Northern Hemisphere countries (e.g., United Kingdom, France, Israel, Italy, the Netherlands, the Philippines, Poland, South Korea, Spain, and the United States) and within one Southern Hemisphere country (Australia) [20].

The public’s acceptance of the influenza vaccine rose during the COVID-19 crisis, resulting in a significant uptick in influenza vaccination rates [12]. Alterations in the public’s inclination towards influenza vaccination could be attributed to several factors, including the following: (1) Perception of personal risk and responsibility. The COVID-19 epidemic has heightened risk awareness as individuals become cognizant of the potential risk of infection to family or friends from preventable diseases and may lack protection against influenza and severe COVID-19 co-infections. This awareness has a positive impact on attitudes toward vaccination. Vaccinated individuals realized the importance of safeguarding themselves, their communities, and at-risk family members to halt the spread of the disease [21]. Specifically, the fear of COVID-19 might be a driving force of influenza vaccination [22]. (2) Mitigating the possibilities of co-infections. As influenza and COVID-19 exhibit similar symptomatic and epidemiological traits, physicians might face difficulty in accurately diagnosing patients during seasonal outbreaks. Thus, an influenza vaccination proves imperative, as it eases the diagnostic process when an individual seeks medical attention, consequently facilitating targeted treatment [18]. (3) Recommendations from healthcare professionals. Healthcare workers are on the frontlines against diseases, realizing the importance of vaccination against infectious respiratory diseases in public prevention. Their vaccination behavior can help the public comprehensively accept the influenza vaccine [23].

Throughout the COVID-19 outbreak, numerous regions adopted non-pharmaceutical interventions (NPIs) via national agencies and local health policies to manage disease epidemics. As a result, influenza activity witnessed a significant downturn from the start of 2020 through to the conclusion of 2021. Nonetheless, renewed viral activity follows when NPIs prove ineffective, spurred by viral mutations, accumulation of vulnerable populations, and increased social events or mass gatherings. Consequently, local health officials and primary care physicians need to cultivate a comprehensive sentinel surveillance network to swiftly identify potential epidemic strains, determine vaccine components, devise vaccination programs, and promote influenza vaccination. Additionally, encouraging local primary care hospitals to partake in symptom and disease reporting via digital devices aids health authorities in forecasting local outbreaks and guiding the diagnosis and treatment process for subsequent patient cases. Public health authorities should ensure frequent publication of surveillance data on suitable platforms to enhance transparency and utilize official social media handles for disseminating accurate information, raising public disease awareness, and dispelling the spread of inaccuracies via unofficial channels. Importantly, high-risk groups, such as the elderly and healthcare workers, hold the key to seasonal influenza management. Governments and decision-makers should debate or enforce mandatory vaccination policies, particularly targeting health sector employees, high-transmission risk industries, and groups susceptible to co-infections to augment seasonal influenza vaccination coverage. Healthcare workers also need to leverage fundamental public health resources to nurture trust with the public, highlight the advantages of influenza vaccination, and bolster the public’s comprehension of vaccine science and confidence in vaccination.

### 4.2. Factors Associated with the Willingness and Practice to Receive Influenza Vaccination

Our research indicates that females can influence individual vaccination willingness and the vaccination behavior of themselves and family members, which is similar to findings from a cross-sectional study conducted in Shanghai and a population-wide study [24,25]. The heightened awareness of health issues and overall well-being among women could illuminate gender disparities in vaccination intentions and historical vaccination statuses [26]. Nonetheless, another cross-sectional study highlighted that mothers demonstrated a higher reluctance than men when considering the influenza vaccination for their children, which is also consistent with our findings [27]. The World Health Organization’s Strategic Advisory Group of Experts (SAGE) describes “vaccine hesitancy” as the postponement or refusal of vaccination services despite their availability [28]. Remarkably, following vaccination, women often exhibit stronger immune responses, endure more adverse reactions, and manifest higher vaccine efficacy, which could potentially discourage women from vaccination.

Moreover, our study revealed a correlation between increasing age and negative vaccination behaviors. Our study found that an advanced age stands as a significant impediment to the willingness to receive an influenza vaccination, which was consistent with a previous study [29]. The elderly may endure financial restrictions, experience physical complications, and harbor suspicions about vaccine safety.

The educational level, number of children, and monthly household income also impacted vaccination intentions and behaviors. Our findings indicated that those with higher education were associated with less willingness to receive the influenza vaccination and exhibited negative vaccination behavior, similar to a cross-sectional study conducted in Spain [30]. Compared with those who only completed primary education, individuals with a university education exhibited lower vaccination willingness. The better-educated population demonstrated increased concerns regarding the safety and efficacy of the vaccines, contributing to vaccine hesitancy. On the contrary, people with lower levels of education had better perceptions and better vaccination coverage. In China, Class I vaccines elicit trust due to the government’s enforcement, but the optional status of Class II vaccines, like influenza vaccines, makes them seem unnecessary to the public [27,31]. Furthermore, those with higher education may overrate their knowledge, fostering self-satisfaction about vaccines [32]. The number of children under fourteen in the family improved vaccination behaviors, potentially due to first-time parents’ hesitations about vaccination [33]. Conversely, families with fewer children revealed a greater aversion and concern toward vaccines due to parents’ insufficient experience [34]. Our study also observed that a higher monthly household income reduced the rates of childhood influenza vaccinations, aligning with another cross-sectional study, where lower-income households exhibited higher child vaccination rates than those with a higher income, potentially due to greater social and material resources and attentive parenting [24].

We encourage vaccination services to become more available and convenient during influenza outbreaks by bolstering educational endeavors related to vaccine health and offering free vaccinations. This strategy presents an opportunity to reduce influenza and flu vaccine hesitancy among highly educated parents and to cater to lower-income families who are reluctant to vaccinate due to logistical constraints.

Improving influenza vaccination rates could also pivot around household incomes. Lower-income households might grapple with vaccine accessibility due to financial restraints, reflecting a higher non-vaccination demographic within this group. Interestingly, our study suggests that parents within these lower-income households might be more open to influenza vaccinations compared to those with higher incomes. Implementing complimentary vaccination services for children in these families can address the accessibility problem, potentially remove vaccination hesitancy within high-income families, and promote widespread inoculation against influenza.

The safety, efficacy, and accessibility of the influenza vaccine are influential determinants of the public’s willingness to accept vaccination. Advancing the development of safe and effective vaccines, promoting their safety and efficiency, cultivating more vaccination points in Guangzhou, educating the public about the side effects, eliminating the public’s fear of these side effects, and rectifying misconceptions related to the vaccine are instrumental in engendering broader acceptance of influenza vaccination.

In Guangzhou, parents’ understanding of the influenza vaccine on average is not optimal, proving influential regarding the prevalence of influenza vaccination. Our study indicated a positive correlation between greater vaccine comprehension, motivation for vaccination, and a progressive attitude toward vaccination, which was consistent with prior cross-sectional studies [35,36,37]. Greater knowledge tends to be associated with healthier behaviors, with vaccinated individuals possessing a deeper understanding of the vaccine than those unvaccinated [38]. Notably, a survey revealed that healthcare workers might also refuse vaccination, citing insufficient vaccine knowledge, which could be reduced by offering on-site vaccination and personalized reminders in outpatient care settings to raise coverage [39]. Perceptions regarding vaccine safety, side effects, and uncertainty about post-marketing effects act as key determinants of vaccination decisions [40,41]. This study found a prevalent mistrust concerning the vaccine’s safety, reducing the propensity for vaccination and encouraging negative vaccination behavior. Indeed, 59.50% refrained from receiving the new vaccine due to doubts surrounding its efficacy, and 56.96% declined due to apprehensions surrounding potential side effects resulting from the vaccine’s swift market introduction. Essentially, the safety, efficacy, and accessibility of the influenza vaccine are fundamental arbiters of vaccination willingness. Therefore, expedited research and development of secure, efficacious influenza vaccines, combined with increased public awareness on vaccine safety and efficiency, along with extended influenza vaccination site availability in Guangzhou, teaching the public about vaccine side effects accurately, allaying fears regarding side effects, and rectifying common vaccine misperceptions could substantially enhance measures to prevent influenza among the public.

Despite yielding valuable insights, this study acknowledges limitations. Primarily, it utilized a cross-sectional design and, due to the inherent methodological constraints, could not establish a definitive causality between willingness for influenza vaccination and potential influencing factors. This study employed snowball convenience sampling rather than rigorously standardized probability sampling, which might have resulted in a non-representative sample. Also, having a reliance on subjectively recalled directly obtained participant responses may have induced recall bias or misinterpretations. Furthermore, there may be selection bias in this study, given the higher proportion of females. Due to the insufficient knowledge items in the questionnaire, the knowledge score is based solely on four responses. Then, the information on the willingness rates of influenza vaccination in the pre-pandemic COVID-19 was not collected, so we cannot compare the rates of willingness in different periods in the same population. Nevertheless, a substantially large sample size reasonably mitigates these limitations. Importantly, this study offers initial insights into the Guangzhou population’s intentions concerning influenza vaccination and may supplement health policy decision-making to a meaningful extent.

## 5. Conclusions

These findings provide essential insights for altering the perceptions of influence as well as influenza vaccination intentions and for enhancing health communication strategies to improve influenza vaccine uptake among Guangzhou residents.

## Figures and Tables

**Table 1:** The general characteristics of participants according to a willingness to accept influenza vaccine.

Characteristics	N (%)	Individuals Willingness to Accept Influenza Vaccine		*χ* ^2^	*p*-Value
		Yes	No		
Socio-demographic characteristics of participants					
Region					
Suburban	3340 (25.28)	1460 (25.93)	1880 (24.80)	2.193	0.139
Urban	9873 (74.72)	4171 (74.07)	5702 (75.20)		
Gender					
Male	3411 (25.82)	1637 (29.07)	1774 (23.40)	54.313	**<0.001**
Female	9802 (74.18)	3994 (70.93)	5808 (76.60)		
Age					
18–29 years	771 (5.84)	386 (6.85)	385 (5.08)	32.500	**<0.001**
30–39 years	5336 (40.38)	2351 (41.75)	2985 (39.37)		
40–49 years	6393 (48.38)	2600 (46.17)	3793 (50.03)		
50 years and older	713 (5.40)	294 (5.23)	419 (5.52)		
Educational level					
Middle school and below	2320 (17.56)	1007 (17.88)	1313 (17.32)	12.608	**0.013**
High school	2185 (16.54)	988 (17.55)	1197 (15.79)		
Specialized training school	3036 (22.98)	1239 (22.00)	1797 (23.70)		
Undergraduate	4769 (36.09)	1998 (35.48)	2771 (36.55)		
Postgraduate and above	903 (6.83)	399 (7.09)	504 (6.64)		
Were you involved in the prevention and control of COVID-19?					
Yes	1891 (14.31)	1025 (18.20)	866 (11.42)	121.156	**<0.001**
No	11,322 (85.69)	4606 (81.80)	6716 (88.58)		
Family monthly income					
<4000	2359 (17.85)	1023 (18.17)	1336 (17.62)	5.570	0.134
4000–9999	4557 (34.49)	1991 (35.36)	2566 (33.84)		
10,000–19,999	3510 (26.56)	1459 (25.91)	2051 (27.05)		
20,000 or more	2787 (21.09)	1158 (20.56)	1629 (21.49)		
Number of family members					
1–3	3722 (28.17)	1465 (26.02)	2257 (29.77)	26.869	**<0.001**
4–5	7480 (56.61)	3242 (57.57)	4238 (55.90)		
Six or more family members	2011 (15.22)	924 (16.41)	1087 (14.33)		
Number of children under fourteen years old in the family					
0	2129 (16.11)	831 (14.76)	1298 (17.12)	19.184	**<0.001**
1	5715 (43.25)	2412 (42.83)	3303 (43.56)		
Two or more children	5369 (40.64)	2388 (42.41)	2981 (39.32)		

Bold: *p-Value* < 0.05.

**Table 2:** Participants’ knowledge and perception of influenza and seasonal influenza vaccine according to a willingness to accept influenza vaccine.

Characteristics	N (%)	Individuals Willingness to Accept Influenza Vaccine		*χ* ^2^	*p*-Value
		Yes	No		
Can influenza vaccine prevent the flu?					
Yes	11,966 (90.56)	5415 (96.16)	6551 (86.40)	360.280	**<0.001**
No	1247 (9.44)	216 (3.84)	1031 (13.60)		
Does the influenza vaccine need to be given every year?					
Yes	8077 (61.13)	4334 (76.97)	3743 (49.37)	1035.881	**<0.001**
No	5136 (38.87)	1297 (23.03)	3839 (50.63)		
Which population should be prioritized for influenza vaccination?					
Wrong answer	8803 (66.62)	3327 (59.08)	5476 (72.22)	250.897	**<0.001**
Correct answer	4410 (33.38)	2304 (40.92)	2106 (27.78)		
Which time of the year is the best time to get a flu shot in Guangzhou?					
Wrong answer	9122 (69.04)	3861 (68.57)	5261 (69.39)	1.019	0.313
Correct answer	4091 (30.96)	1770 (31.43)	2321 (30.61)		
Do you think the influenza vaccine is safe?					
It’s safe, basically no side effects	3773 (28.56)	2123 (37.70)	1650 (21.76)	467.852	**<0.001**
Basically safe, with a few side effects	9192 (69.57)	3478 (61.77)	5714 (75.36)		
It’s not safe. Side effects are obvious.	248 (1.87)	30 (0.53)	218 (2.88)		
Knowledge score					
Full marks	1093 (9.02)	694 (12.32)	399 (5.26)	212.385	**<0.001**
Wrong answers or omissions	12,120 (90.98)	4937 (87.68)	7183 (94.74)		
Total	13213	5631 (42.62)	7582 (53.38)		

Bold: *p-Value* < 0.05.

**Table 3:** Previous influenza vaccination of participants and their families.

Potential Factors	If Children and Teenagers Used to Receive Influenza Vaccine		χ^2^	*p*-Value	If Participants or Other Family Members (Not Including the Children and Teenagers) Used to Receive Influenza Vaccine		χ^2^	*p*-Value
	Yes	No			Yes	No		
Socio-demographic characteristics of parents, their children, and teenagers								
Region								
Suburban	2031 (27.75)	1309 (22.21)	52.917	**<0.001**	1381 (25.85)	1959 (24.89)	1.536	0.215
Urban	5289 (72.25)	4584 (77.79)			3962 (74.15)	5911 (75.11)		
Gender								
Male	1939 (26.49)	1472 (24.98)	3.888	0.049	1576 (29.50)	1835 (23.32)	63.469	**<0.001**
Female	5381 (73.51)	4421 (75.02)			3767 (70.50)	6035 (76.68)		
Age								
18–29 years	427 (5.83)	344 (5.84)	40.162	**<0.001**	358 (6.70)	413 (5.25)	18.781	**<0.001**
30–39 years	3121 (42.64)	2215 (37.59)			2206 (41.29)	3130 (39.77)		
40–49 years	3419 (46.71)	2974 (50.47)			2494 (46.68)	3899 (49.54)		
50 years and older	353 (4.82)	360 (6.10)			285 (5.33)	428 (5.44)		
Education level								
Middle school and below	1476 (20.16)	844 (14.32)	213.724	**<0.001**	1022 (19.13)	1298 (16.49)	27.468	**<0.001**
High school	1378 (18.83)	807 (13.69)			907 (16.98)	1278 (16.24)		
Specialized training school	1683 (22.99)	1353 (22.96)			1135 (21.24)	1901 (24.16)		
Undergraduate	2374 (32.43)	2395 (40.64)			1935 (36.22)	2834 (36.01)		
Postgraduate and above	409 (5.59)	494 (8.39)			344 (6.43)	559 (7.1)		
Were you involved in the prevention and control of COVID-19?								
Yes	1041 (14.22)	850 (14.42)	0.109	0.741	918 (17.18)	973 (12.36)	60.238	**<0.001**
No	6279 (85.78)	5043 (85.58)			4425 (82.82)	6897 (87.64)		
Family monthly income								
<4000	1499 (20.48)	860 (14.59)	137.900	**<0.001**	1007 (18.85)	1352 (17.18)	8.385	**0.039**
4000–9999	2642 (36.09)	1915 (32.50)			1859 (34.79)	2698 (34.28)		
10,000–19,999	1789 (24.44)	1721 (29.20)			1383 (25.88)	2127 (27.03)		
20,000 or more	1390 (18.99)	1397 (23.71)			1094 (20.48)	1693 (21.51)		
Number of family members								
1–3	1919 (26.22)	1803 (30.60)	31.098	**<0.001**	1365 (25.55)	2357 (29.95)	30.924	**<0.001**
4–5	4249 (58.05)	3231 (54.83)			3122 (58.43)	4358 (55.37)		
Six or more family members	1152 (15.73)	859 (14.57)			856 (16.02)	1155 (14.68)		
Number of children under fourteen years old in family								
0	1004 (13.72)	1125 (19.09)	74.79	**<0.001**	717 (13.42)	1412 (17.94)	51.212	**<0.001**
1	3198 (43.69)	2517 (42.71)			2340 (43.80)	3375 (42.88)		
Two or more children	3118 (42.59)	2251 (38.20)			2286 (42.78)	3083 (39.18)		
Knowledge and perception towards influenza and seasonal influenza vaccine of parents								
Can influenza vaccine prevent the flu?								
Yes	6811 (93.05)	5155 (87.48)	118.498	**<0.001**	5011 (93.79)	6955 (88.37)	109.087	**<0.001**
No	509 (6.95)	738 (12.52)			332 (6.21)	915 (11.63)		
Does the influenza vaccine need to be given every year?								
Yes	4852 (66.28)	3225 (54.73)	183.550	**<0.001**	3694 (69.14)	4383 (55.69)	242.096	**<0.001**
No	2468 (33.72)	2668 (45.27)			1649 (30.86)	3487 (44.31)		
Which population should be prioritized for influenza vaccination?								
Wrong answer	4745 (64.82)	4058 (68.86)	23.951	**<0.001**	3349 (62.68)	5454 (69.30)	62.739	**<0.001**
Correct answer	2575 (35.18)	1835 (31.14)			1994 (37.32)	2416 (30.70)		
Which time of the year is the best time to get a flu shot in Guangzhou?								
Wrong answer	5021 (68.59)	4101 (69.59)	1.522	0.217	3705 (69.34)	5417 (68.83)	0.390	0.532
Correct answer	2299 (31.41)	1792 (30.41)			1638 (30.66)	2453 (31.17)		
Do you think the influenza vaccine is safe?								
It’s safe, basically no side effects	2569 (35.10)	1204 (20.43)	384.073	**<0.001**	1886 (35.30)	1887 (23.98)	217.349	**<0.001**
Basically safe, with a few side effects	4675 (63.87)	4517 (76.65)			3398 (63.60)	5794 (73.62)		
It’s not safe. Side effects are obvious.	76 (1.03)	172 (2.92)			59 (1.10)	189 (2.40)		
Knowledge score								
Full marks	693 (9.47)	400 (6.79)	30.891	**<0.001**	546 (10.22)	547 (6.95)	44.807	**<0.001**
Wrong answers or omissions	6627 (90.53)	5493 (93.21)			4797 (89.78)	7323 (93.05)		
Total	7320 (55.40)	5893 (44.60)			5343 (40.44)	7870 (59.56)		

Bold: *p-Value* < 0.05.

**Table 4:** Factors associated with the willingness and previous behavior of influenza vaccination.

Potential Factors	Individuals’ Willingness to Accept Influenza Vaccine (Model 1)		Children and Teenagers Who Used to Receive Influenza Vaccines (Model 2)		Participants or Other Family Members (Not Including Children and Teenagers) Used to Receive Influenza Vaccine (Model 3)	
	OR (95% CI)	*p*-Value	OR (95% CI)	*p*-Value	OR (95% CI)	*p*-Value
Socio-demographic factors						
Region						
Suburban	NA	NA	1.00		NA	NA
Urban	NA	NA	0.925 (0.846,1.011)	0.084	NA	NA
Gender						
Male	1.00		1.00		1.00	
Female	1.395 (1.278,1.522)	**<0.001**	0.911 (0.837,0.991)	**0.030**	1.366 (1.257,1.485)	**<0.001**
Age						
18–29 years	1.00		1.00		1.00	
30–39 years	0.782 (0.665,0.920)	**0.003**	1.164 (0.994,1.362)	0.059	0.811 (0.694,0.948)	**0.008**
40–49 years	0.726 (0.617,0.853)	**<0.001**	1.033 (0.883,1.208)	0.684	0.788 (0.674,0.920)	**0.003**
50 years and older	0.753 (0.599,0.945)	**0.015**	0.879 (0.706,1.094)	0.247	0.857 (0.688,1.068)	0.169
Education level						
Middle school and below	1.00		1.00		1.00	
High school	1.038 (0.915,1.178)	0.560	1.036 (0.910,1.179)	0.591	0.875 (0.772,0.991)	**0.036**
Specialized training school	0.886 (0.787,0.998)	**0.046**	0.798 (0.704,0.906)	**<0.001**	0.764 (0.675,0.864)	**<0.001**
Undergraduate	0.858 (0.768,0.959)	**0.007**	0.649 (0.570,0.738)	**<0.001**	0.847 (0.747,0.959)	**0.009**
Postgraduate and above	0.872 (0.736,1.034)	0.115	0.534 (0.444,0.642)	**<0.001**	0.713 (0.594,0.857)	**<0.001**
Were you involved in the prevention and control of COVID-19?						
No	1.00		NA	NA	1.00	
Yes	1.551 (1.396,1.724)	**<0.001**	NA	NA	1.362 (1.231,1.507)	**<0.001**
Family monthly income						
<4000	NA	NA	1.00		1.00	
4000–9999	NA	NA	0.895 (0.800,1.001)	0.053	0.990 (0.887,1.105)	0.853
10,000–19,999	NA	NA	0.761 (0.670,0.864)	**<0.001**	0.946 (0.834,1.072)	0.384
20,000 or more	NA	NA	0.795 (0.693,0.912)	**0.001**	0.970 (0.846,1.111)	0.658
Number of family members						
1–3	1.00		1.00		1.00	
4–5	1.078 (0.978,1.187)	0.131	1.012 (0.922,1.110)	0.804	1.093 (0.996,1.200)	0.062
Six or more family members	1.132 (0.987,1.298)	0.077	0.941 (0.824,1.075)	0.369	1.065 (0.933,1.216)	0.353
Number of children under fourteen years old in family						
0	1.00		1.00		1.00	
1	0.990 (0.881,1.111)	0.859	1.389 (1.245,1.550)	**<0.001**	1.303 (1.164,1.460)	**<0.001**
Two or more children	0.993 (0.867,1.137)	0.919	1.474 (1.295,1.678)	**<0.001**	1.336 (1.171,1.525)	**<0.001**
Knowledge and perception toward influenza and seasonal influenza vaccine aspects						
Can influenza vaccine prevent the flu?						
No	1.00		1.00		1.00	
Yes	2.474 (2.106,2.906)	**<0.001**	1.438 (1.265,1.635)	**<0.001**	1.509 (1.313,1.734)	**<0.001**
Does the influenza vaccine need to be given every year?						
No	1.00		1.00		1.00	
Yes	2.756 (2.540,2.992)	**<0.001**	1.369 (1.268,1.478)	**<0.001**	1.523 (1.408,1.648)	**<0.001**
Which population should be prioritized for influenza vaccination?						
Wrong answer	1.00		1.00		1.00	
Correct answer	1.464 (1.343,1.596)	**<0.001**	1.136 (1.045,1.235)	**0.003**	1.224 (1.126,1.331)	**<0.001**
Do you think the influenza vaccine is safe?						
It’s safe, basically no side effects	1.00		1.00		1.00	
Basically safe, with a few side effects	0.585 (0.539,0.635)	**<0.001**	0.572 (0.526,0.621)	**<0.001**	0.681 (0.629,0.737)	**<0.001**
It’s not safe. Side effects are obvious.	0.284 (0.188,0.429)	**<0.001**	0.320 (0.238,0.430)	**<0.001**	0.526 (0.384,0.722)	**<0.001**
Knowledge score						
Full marks	1.00		1.00		1.00	
Wrong answers or omissions	0.813 (0.701,0.942)	**0.006**	0.824 (0.711,0.955)	**0.010**	0.922 (0.799,1.063)	0.264

Model 1 assessed the associations between socio-demographic factors, knowledge and perception factors toward influenza and seasonal influenza vaccine, and individuals’ willingness to accept the vaccine. Model 2 assessed the associations between socio-demographic factors, knowledge and perception factors toward influenza and seasonal influenza vaccine, and the children’s previous history of vaccination. Model 3 assessed the associations between socio-demographic factors, knowledge and perception factors toward influenza and seasonal influenza vaccine, and the individuals and their family members’ previous history of vaccination. NA is “not applicable” because only the statistically significant variables are involved in the corresponding regression model. Bold: *p-Value* < 0.05.

## Data Availability

The data in this article was unavailable due to copyright consideration and privacy.

## Supplementary Materials

The following supporting information can be downloaded at: https://www.mdpi.com/article/10.3390/vaccines12101169/s1, Table S1. Knowledge score; Table S2. The questionnaire of influenza vaccine and vaccination; Table S3. Status of knowledge score.
