# Supplementary material for: Knowledge, Attitudes, and Practices towards Influenza Vaccine among Guangzhou Residents: A Cross-Sectional Study
Source: Vaccines (Basel). 2024 Oct 14;12(10):1169. doi: 10.3390/vaccines12101169 (PMC11511243; doi:10.3390/vaccines12101169)
Supplement: Supplementary file 1 [file vaccines-12-01169-s001.zip › vaccines-3189325-supplementary.pdf]

Supplementary Table S1 Knowledge score

Questions	Options	Answer	Score
Can influenza vaccine prevent the flu?	Yes	Correct answer	1
	No	Wrong answer	0
Does the influenza vaccine need to be given every year?	Yes	Correct answer	1
	No	Wrong answer	0
Which population should be prioritized for influenza vaccination?	Children aged six months to five years<br>Aged 60 and older<br>Patients with chronic diseases<br>Healthcare workers<br>Family members and caregivers of infants under six months of age	Choosing all options were considered to be correct	1
	-	Omitted as incorrectly answered	0
Which time of the year is the best time to get a flu shot in Guangzhou?	1-4 months	Wrong answer	0
	5-8 months	Wrong answer	0
	9-12 months	Correct answer	1
	All year	Wrong answer	0

Supplementary Table S2 The questionnaire of influenza vaccine and vaccination

Questions	options	N	%
Participants' knowledge and perception toward influenza vaccination			
Can influenza vaccine prevent the flu?	No	1247	9.44
	Yes	11966	90.56
Does the influenza vaccine need to be given every year?	No	5136	38.87
	Yes	8077	61.13
Which population should be prioritized for influenza vaccination? (multiple choices)	Children aged six months to five years	11286	85.42
	Aged 60 and older	9925	75.12
	Patients with chronic diseases	6440	48.74
	Healthcare workers	8143	61.63
	Family members and caregivers of infants under six months of age	7240	54.79
	Choose all (answered correctly)	4410	33.38
Which time of the year is the best time to get a flu shot in Guangzhou?	1-4 months	3119	23.61
	5-8 months	718	5.43
	9-12 months	4091	30.96
	All year	5285	40.00

Do you think the influenza vaccine is safe?	It's safe, basically no side effects	3773	28.56
	Basically safe, with a few side effects	9192	69.57
	It's not safe. Side effects are obvious.	248	1.88
Participants' willingness and attitude toward influenza vaccination			
Willingness of participants and their family members (not including child) toward influenza vaccination (N=13213)	Yes	5631	42.62
	No	7582	57.38
Reasons for participants and their family members (not including child) to consider getting the flu vaccine (multiple choices) (N=5631)	The COVID-19 epidemic is serious and the fear of infection affects the normal life of the individuals	5011	88.99
	Group vaccination organized by the workplace	1017	18.06
	Knowing that friends and relatives in the neighborhood have vaccination plans	1555	27.61
	Getting the flu shot for fear of contracting the COVID-19	1932	34.31
Willingness of starting to take their own kids for a flu shot in this year (N=5891)	Yes	2860	48.55
	No	3031	51.45
Reasons to consider bringing your child for a flu shot (multiple choices)	Fears of infection affecting children and their families as COVID-19 outbreak	2527	88.36

(N=2860)	becomes serious		
	Everyone we know has their kids vaccinated	341	11.92
	School, community, and doctor recommendations	963	33.67
Reasons to not to consider bringing your child for a flu shot (multiple choices) (N=3031)	Too expensive	352	11.61
	Influenza is not a serious illness, don't think it's necessary	985	32.50
	Concerns about security	1613	53.22
	I've heard that the results are mediocre	979	32.30
	Don't know where to vaccination	366	12.08
Reasons for taking children for vaccination in the past (N=7322)	School Requirements	1380	18.85
	Doctor's Recommendations	2415	32.98
	Spontaneous vaccination	3325	45.41
	Family Recommendations	202	2.76
Willingness of continuing to take their own kids for influenza vaccine in the next year （N=7332）	Yes	4588	62.66
	No	2734	37.34
Reasons for continuing vaccination of children (multiple choices) (N=4588)	Very effective, vaccination of children rarely catch a cold	2168	47.25
	Can prevent other members of the family be infected	2667	58.13

	Everyone we know has their kids vaccinated	656	14.30
	Affected by the COVID-19 epidemic, just in case	2478	54.01
Reasons for not continuing vaccination of children (multiple choices) (N=2734)	The effect is not significant, just the same as the probability of catching a cold	1697	62.07
	Too expensive	341	12.47
	Too many side effects, not safe enough	407	14.89
	Many places can't get the flu vaccine, and it's too much troubles to get a single shot	826	30.21
Previous vaccination status of children, parents, and their family members in the past three months			
Has your child ever had a influenza vaccine? (N=13213)	Yes	7322	55.42
	No	5891	44.58
Frequency of influenza vaccination for children (N=7322)	Once a year	2199	30.03
	Every few years	2468	33.71
	Only once	2655	36.26
Have other family members (not including child) or yourself used to get influenza vaccine? (N=13213)	Yes	5343	40.44
	No	7870	59.56

Supplementary Table S3 Status of knowledge score

Knowledge score	N	%
0	518	3.92
1	2851	21.58
2	4930	37.31
3	3821	28.92
4	1093	8.27
